# The Interplay of Glycosaminoglycans and Cysteine Cathepsins in Mucopolysaccharidosis

**DOI:** 10.3390/biomedicines11030810

**Published:** 2023-03-07

**Authors:** Alexis David, Thibault Chazeirat, Ahlame Saidi, Gilles Lalmanach, Fabien Lecaille

**Affiliations:** 1 Faculty of Medicine, University of Tours, F-37032 Tours, France; 2 Team “Proteolytic Mechanisms in Inflammation”, INSERM, UMR1100, Research Center for Respiratory Diseases (CEPR), F-37032 Tours, France

**Keywords:** cysteine proteases, glycosaminoglycans, inhibition, mucopolysaccharidosis, proteolysis

## Abstract

Mucopolysaccharidosis (MPS) consists of a group of inherited lysosomal storage disorders that are caused by a defect of certain enzymes that participate in the metabolism of glycosaminoglycans (GAGs). The abnormal accumulation of GAGs leads to progressive dysfunctions in various tissues and organs during childhood, contributing to premature death. As the current therapies are limited and inefficient, exploring the molecular mechanisms of the pathology is thus required to address the unmet needs of MPS patients to improve their quality of life. Lysosomal cysteine cathepsins are a family of proteases that play key roles in numerous physiological processes. Dysregulation of cysteine cathepsins expression and activity can be frequently observed in many human diseases, including MPS. This review summarizes the basic knowledge on MPS disorders and their current management and focuses on GAGs and cysteine cathepsins expression in MPS, as well their interplay, which may lead to the development of MPS-associated disorders.

## 1. Introduction

Lysosomal storage diseases (LSDs) are a large group of over seventy metabolic disorders such as Pompe disease, Gaucher disease, Fabry disease, the Niemann–Pick disorders, and mucopolysaccharidosis (MPS) and are caused by inherited gene mutations that alter lysosomal homeostasis [1]. Lysosomal enzymes are affected the most, and deficiency in them results in a progressive accumulation of specific macromolecules inside the endosomal–autophagic–lysosomal system.

MPS is a group of seven inborn genetic disorders and is characterized by an inherent deficiency of lysosomal enzymes that are responsible for the breakdown of specific glycosaminoglycans (GAGs). The abnormal storage process leads to a broad spectrum of adverse health outcomes depending on GAGs levels and location, contributing progressively to morbidity and early mortality. 

Over the past two decades, numerous studies have demonstrated a beneficial effect in the existing treatments for LSDs, including MPS [2]. Nevertheless, despite considerable success in reducing morbidity and improving the quality of life of some MPS patients, current therapies are unable to cure all clinical manifestations such as neurological, skeletal, and cardiorespiratory symptoms. Future treatment options such as targeted gene therapy (TGT), anti-inflammatory therapy, and substrate reduction therapy are currently under experimental stages and their outcomes need to be validated in human trials [3]. 

MPS pathophysiology emanates not only from the direct effects of elevated GAG storage but also is the result of a complex cascade of secondary events in cells with an intricate interplay, contributing to the dysfunction of affected tissues and the complexity of MPS. Thus, exploring new treatments based on the molecular mechanisms and pathological changes underlying MPS is imperative. Lysosomes are acidic subcellular compartments that play a central role in the turnover and recycling of diverse substrates (e.g., endocytosis products, macromolecules from damaged cells organelles). Beside lipases, glycosidases, nucleases, sulfatases or phosphatases, and several families of proteases, including cysteine cathepsins, are found in lysosomes. Cysteine cathepsins were reported to play a key role for several physiological functions. Accordingly, a dysregulation of their expression and/or their proteolytic activity can lead to the development of various human pathologies, including MPS [4,5]. Considering their role in pathological processes, inhibition of specific cathepsins has become an attractive therapeutic strategy. In addition, the activity of cysteine cathepsins can be controlled in several ways, GAGs (depending on their nature and levels) being the foremost one for some cathepsins [6].

In this review, we summarize the main characteristics and management of MPS and focus on GAGs and cysteine cathepsins expressions in MPS and their regulation by GAGs, which may have consequences for MPS pathogenesis. 

## 2. Mucopolysaccharidosis

### 2.1. Incidence, Clinical Features, and Diagnosis

MPS represents a group of rare and inherited lysosomal storage diseases that are clinically heterogeneous and characterized by multiorgan involvement due to the accumulation of mucopolysaccharides, aka glycosaminoglycans (GAGs), at the lysosomal level, resulting in reduced life expectancy. The enzymes involved in the degradation of GAG, including heparan sulfate (HS), dermatan sulfate (DS), chondroitin sulfate (CS), keratan sulfate (KS), and/or hyaluronic acid (HA), singly or in combination, are deficient (absence or malfunction) in these pathologies. Depending on the deficient enzyme, seven types of MPS syndromes designated MPS-I to MPS-IX (with the exclusion of MPS-V and MPS-VIII, which are no longer used) with 13 subtypes are reported (Table 1). 

MPS are orphan diseases with an incidence estimated to range from 1 per 25,000 and 1 per 100,000 live births, depending on the MPS type [7]. The incidence of MPS types may also be related to continent and ethnic background [8]. The first cases of MPS were described by Charles Hunter in 1917 [9], and two years later, MPS-I cases were reported by Hurler. MPS-IX is the rarest form of mucopolysaccharidosis, with only four patients diagnosed to date [10]. MPS are autosomal recessive genetic diseases, except for MPS type II, which is an X-linked genetic disease [11]. Consequently, lysosomal GAGs accumulate progressively in various tissues, and partially degraded GAGs are excreted in urine. Abnormal GAG storage triggers a cascade of cellular events and progressively prompts organ dysfunction. Clinical features depend on the specific enzyme deficiency and the organs affected by GAGs. While not visible at birth, the first clinical symptoms appear during early childhood. Clinical symptoms are mainly coarse facial features, connective and bone damage, cardiac, respiratory, hearing, and vision disorders, and in most cases, mental retardation [12]. Symptoms may be similar or vary among the different MPS. Clinical examination and several qualitative and quantitative tests (e.g., Elisa, dye-spectrometric, thin layer chromatography, electrophoresis, LC-MS/MS methods) to evaluate GAG levels in urine are the first steps in the diagnosis of a MPS disease [13,14,15,16,17]. For parents with a family history of MPS, a prenatal diagnosis procedure is possible using amniocentesis and chorionic villus sampling to detect whether the fetus is carrying the mutated gene (Table 1).

### 2.2. Management

At present, there is no effective curative treatment that can restore mutated genes of patients with MPS. However, depending on the degree of severity and timely diagnosis, different therapeutic options are possible. Current clinical practices such as HSCT and ERT are dedicated to mitigating the progression of MPS and improving the quality of life of patients [18]. Novel experimental therapies for MPS such as gene therapy (GT), anti-inflammatory therapy, substrate reduction therapy (SRT), and pharmacological chaperone therapy have been investigated and may represent promising avenues. To date, beside supportive or symptomatic care that can improve the quality of life for patients and parents, no effective therapy is yet approved for MPS-III patients (Table 1). 

The understanding of mucopolysaccharidosis has been facilitated by the study of animal models, which naturally present the same phenotypes as humans due to mutations in orthologous genes. These animals are often domestic species, especially dogs and cats. Over the past few decades, knockout mouse models with phenotypes similar to the different types of MPS have emerged [19].

#### 2.2.1. Hematopoietic Stem Cell Transplantation 

HSCT involves a blood cell transplant of donor cells from three different sources: bone marrow, peripheral blood stem cells, and umbilical cord blood (for review: [20]). The monocyte–macrophage system is the basic mechanism of therapeutic action, as it relies on the ability of circulating monocytes to escape from vessels and migrate inside organs where they transform into macrophages. When macrophages reach the different sites, they secrete the functional enzyme, which is internalized by the surrounding affected cells; the enzyme then reaches the lysosomes and degrades the stored and undigested material. However, as this process is slow and incomplete, success is limited for the treatment of severe neurological diseases. Although very few studies have used this approach, HSCT has been shown to increase life expectancy and improve clinical manifestation in children with attenuated Hurler disease when performed early in life and with preparative conditioning regimens to reduce graft–host disease, infection, and additional complications [21]. For other types of MPS, HSCT has not had the same success as for Hurler’s disease, and other therapeutic approaches have been developed.

#### 2.2.2. Enzyme Replacement Therapy 

In 1964, Christian de Duve first suggested that ERT might be a therapeutic option to treat lysosomal storage diseases [22]. ERT has been approved for MPS-I (2003), MPS-II (2006), MPS-IVA (2015), MPS-VI (2005), and MPS-VII (2017). The aim of ERT is to compensate metabolic defects in MPS patients by weekly or fortnightly infusions of recombinant enzymes. Contrary to HSCT, in which the functional enzyme is expressed and circulated indefinitely, enzyme experiences in ERT display a rapid clearance and a half-life, typically under 1 h. The clinical efficacy is highly variable depending on the health status of the patients. Moreover, it is very difficult to target the recombinant enzymes on tissues that are not easily accessible by the systemic circulation, in particular bones, cartilages, or brain (blood–brain barrier), although the search for new and more effective therapeutic strategies is in progress [23,24,25,26]. Alternatively, the use of ERT in combination with HSCT may present significant therapeutic benefits by the possibility to resolve immune response and reduce symptoms and decrease mortality rates [20].

#### 2.2.3. Gene Therapy

The success of this approach has been demonstrated in several MPS animal models [27]. Phase I/II clinical trials are underway for MPS-I, -II, -IIIA, -IIIB, and -VI in several countries (for review: [28]). It involves either in vivo therapy, with the direct injection of therapeutic gene intravenously or locally to target somatic cells through an appropriate viral or non-viral vector, and ex vivo therapy, in which the vector is transfected into somatic cells derived from MPS patient and then re-administrated into the recipient. Transduced cells should be able to continuously secrete supra-physiological enzyme levels in all organs affected by MPS. As the secreted enzyme cannot cross the blood–brain barrier, the benefits of this approach are generally limited to peripheral organs, although recent studies have also shown the efficiency of gene transmission and expression after injection directly into the central nervous system (CNS) (for review: [29]). A phase I/II clinical trial has been launched in patients with type IIIA MPS [30]. The delivery of sufficient enzyme in CNS and bone, the high immunogenic toxicity of both vectors and transgene, and the relatively high cost of this technology remain an unmet challenge for GT [31].

#### 2.2.4. Anti-Inflammatory Drugs, Substrate Reduction, and Pharmacological Chaperone Therapeutic Strategies

In the last decade, new therapeutic options have been investigated for MPS patients and are under clinical trials such as anti-inflammatory drugs, substrate reduction therapy (SRT), and pharmacological chaperone therapy. To suppress metabolic inflammation caused by GAGs accumulation, anti-inflammatory treatments in combination with current MPS treatment could be an alternative to inhibit secreted cytokines using blocking antibodies, impair cell–cell interactions, or suppress specific cell types [32,33,34]. Substrate reduction therapy (SRT) is another therapeutic approach, which consists of directly or indirectly slowing down GAG biosynthesis with the use of small inhibitors to reduce lysosomal storage. Contrary to ERT, these small molecules can cross the blood–brain barrier and have the potential to directly treat CNS symptoms of MPS. Preclinical and clinical trials, however, showed various outcomes [35,36], and SRT has not been approved yet for any MPS. In MPS, deficiencies in enzymes involved in GAG catabolism are due to mutations, which in some cases affect full processing, folding, and lysosomal targeting. Pharmacological chaperone therapy (PCT) aims to use small molecules that specifically bind to the mutated enzyme to enhance its correct folding, stability, and intracellular trafficking [37]. PCTs have the advantage of wide tissue distribution, potential oral distribution, and low immunogenicity. 

## 3. Glycosaminoglycans in MPS

GAGs are a family of highly complex, linear, and heterogeneous polysaccharides that consist of repeating disaccharide units with varying chain length, type of linkage, and extent of sulfation and epimerization. They can be categorized into four main groups: heparin (Hep)/heparan sulfate (HS); chondroitin sulfate (CS)/dermatan sulfate (DS); keratan sulfate (KS); and hyaluronan (HA) [38]. This chapter will only introduce major characteristics of sulfated GAGs and their metabolism. Due to the extreme rarity of MPS-IX patients (only four patients have ever been reported [10]), features of non-sulfated HA will not be detailed here.

### 3.1. Structure, Expression, Catabolism, and MPS Disorders 

GAGs are negatively charged polysaccharide chains with a molecular weight of approximately 10–100 kDa, except for HA, which exhibits molecular weights in the range of 4–8000 kDa (Table 2). Among GAGs, two categories: non-sulfated (HA) and sulfated GAGs (CS, DS, KS, Hep, and HS) can be distinguished. The chains of GAG are composed of repeated disaccharide units, including uronic acid and a hexosamine, except for KS where uronic acid is replaced by galactose (Gal). Uronic acid exists in two forms: iduronic acid (IdoA) or glucuronic acid (GlcA). For hexosamine, it can be either N-acetyl glucosamine (GlcNAc) or N-acetyl galactosamine (GalNAc) [39]. The structural diversity of GAGs is enhanced by different degrees of modification of the disaccharide subunits. Indeed, the hydroxyl groups in position C2 of uronic acid and in positions C3, C4, and C6 of hexosamine can be O-sulphated, and glucosamines can be N-acetylated or N-sulfated (or more rarely N-unsubstituted). Subsequently, an octa-saccharide could exhibit over 1,000,000 different sulfation sequences [40].

#### 3.1.1. Heparin/Heparan Sulfate

**Figure 1:**
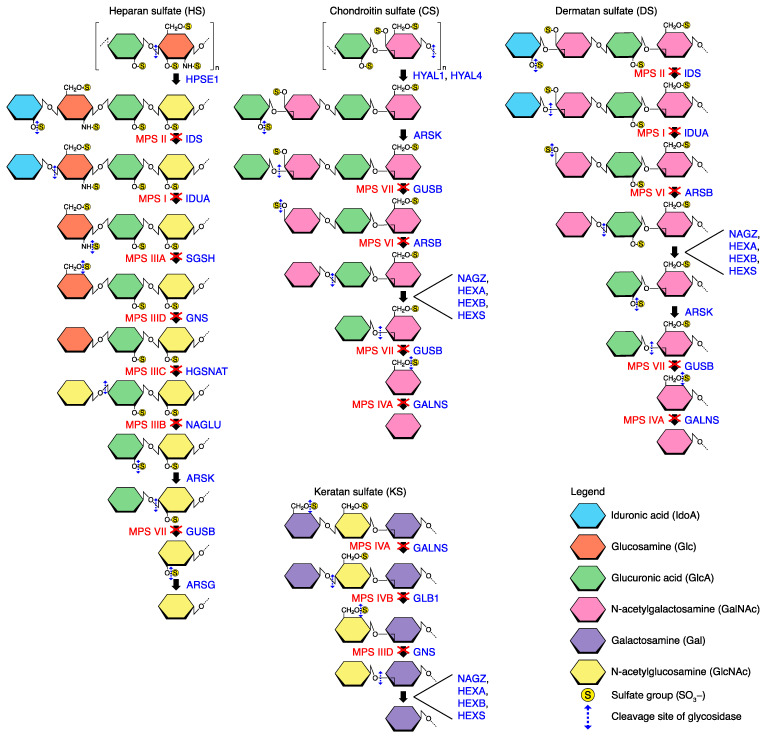
Degradation of the sulfated GAG chains. The deficient enzymes involved in the different MPS subtypes are indicated. HYAL1/4 and HPSE1 are endoglycosydases capable of degrading long chains of CS and HS, respectively, into smaller fragments. Degradation of GAGs consists in repeating steps of desulfation and deglycosylation at the non-reducing end of the chain by sulfatase and exoglycosydase, respectively. Enzymes involved in the catabolism of GAGs and in MPS subtypes associated are indicated. ARSB (arylsulfatase B); ARSG (arylsulfatase G); ARSK (arylsulfatase K); GALNS (galactosamine N-acetyl)-6-sulfatase); GLB1 (β1-galactosidase); GNS (glucosamine (N-acetyl-6-sulfatase)); GUSB (β-glucuronidase); HEXA (hexosaminidase subunit α); HEXB (β-hexosaminidase); HEXS (hexosaminidase S); HGSNAT (heparan-α-glucosaminide N-acetyltransferase); HPSE1 (heparanase); HYAL1/4 (hyaluronidase 1/4); IDS (iduronate 2-sulfatase); IDUA (α-L-iduronidase); NAGLU (N-acetyl-α-glucosaminidase); NAGZ (β-N-acetylglucosaminidase); SGSH (N-sulfoglucosamine sulfohydrolase).

Heparin typically consists of shorter disaccharide repeating units of β1,4-linked α-L-iduronic and α-D-glucosamine, in which the predominant substitution pattern is 2-O-sulfation of the iduronate residues and N- and 6-O-sulfation of the glucosamine residues [43]. Other substitutions including N-acetylation and 3-O-sulfation may be present in glucosamine. In heparan sulfate (HS), uronic acid is predominantly β-D-glucuronic acid, the C5 epimer of α-L-iduronic. HS is naturally present in all cells and varies in terms of degree of sulfation and chain length depending on the biological origin. HS chains are generally made up of 50 to 250 disaccharide units (20 to 100 kDa). At physiological pH, all carboxylic and sulfate functions are deprotonated, giving GAGs high negative charge densities (heparin has the highest negative charge density of any known mammalian GAGs) [44]. Sulfation of the various hydroxyl groups or the amino group present on the glucosamine compound of HS/Hep drives its ability to interact with various proteins, cytokines, and growth factors [45]. While Hep is largely restricted to mast cells, HS is ubiquitously expressed on cell surfaces and in the extracellular matrix (ECM) and basement membrane (BM) in mammalian tissues. HS/Hep are tethered to proteins through a tetra-saccharide linker, covalently bound to a serine residue to form proteoglycans (PGs) (for review: [46]). Heparan sulfate proteoglycans (HSPGs) are classified into three groups according to their location: (i) transmembrane HSPGs, such as syndecans 1–4 (carrying HS and CS chains) and glypicans 1–6 (HS chains), (ii) pericellular and extracellular HSPGs including agrin (HS chains), perlecan (HS chains) and type XV and XVIII collagens (HS chains), and testicans 1–3 (HS chains), and (iii) the secretory vesicle serglycin (Hep and CS chains). Proteoglycans participate in many biological processes such as cell regulation (growth, proliferation, and migration) [47,48], CNS development and repair [49,50], and cell recognition [51,52]. Hurler syndrome, the severe form of MPS-I, is associated with neurological and/or behavioral abnormalities, as observed in MPS-II, -III, and -VII, where HS is accumulated (Figure 1). Since HS is the primary storage material in these MPS types, HS could be an interesting candidate as a biomarker of brain pathology and neurological manifestations for MPS-I, -II, -III, and -VII, by measuring its levels in urine and blood. Treatment of MPS-II mice with a blood–brain-barrier-penetrable antibody (Pabinafusp Alfa) reduces HS levels in brain and prevents neurodegeneration and neurocognitive dysfunction [53]. The accumulation of HS in MPS-I, -II, -III, and -VII affects lysosomal functions, leading to numerous irreversible alterations within and outside cells (e.g., abnormal composition of membranes, intracellular vesicle trafficking, autophagy, mitochondrial dysfunction, oxidative stress, inflammation) [54,55].

Additionally, accumulation of membrane-bound cell-surface HSPGs may alter growth-factor–receptor interactions and signal transduction [56]. Besides neural dysfunctions, HS can lead to the progressive development of a variety of clinical manifestations, including ear, nose, throat, and respiratory problems, which are often the first emerging symptoms in all MPS types [17,57]. Thickened depositions/secretions in airways and interstitium due to an abnormal accumulation of ECM components can further exacerbate lung obstruction. Due to the unsuitability to monitor respiratory function in young MPS patients, an alternative non-invasive method named global respiratory symptoms severity (GRSS) was developed [58]. GRSS is a score ranging from 0 to 4, which relies on four lung restriction subtypes: (i) ear–nose–throat symptoms (chronic rhinitis or sinusitis, otitis, adeno-tonsillar hypertrophy, hearing loss, macroglossia, stridor), (ii) pulmonary symptoms (dyspnea, wheezing, cough, sputum, asthma, bronchitis, pneumonia), (iii) clinical symptoms of obstructive sleep apnea, and (iv) skeletal abnormalities causing restrictive lung disease (scoliosis, kyphosis, ribcage narrowing, chest wall deformity). We reported that HS levels, which is the most abundant GAG in the lungs, increased in respiratory secretions of MPS-I, -II, and -III young patients compared to non-MPS patients, and correlated positively to the severity of respiratory symptoms (GRSS) that worsen with age [58]. 

#### 3.1.2. Chondroitin Sulfate/Dermatan Sulfate

Chondroitin sulfate consists of repeating GlcA-GalNAc disaccharide units linked by β1,3 and β1,4, respectively. Sulfate groups can be either present at the C4 (C4-S or CS-A) or C6 (C6-S or CS-C), or both C4 and C6 (C4,6-S or CS-E) hydroxyl groups of GalNAc units. The GlcA unit can also be sulfated at the C2 position, giving rise to CS-D (6-sulfated GalNAc and 2-sulfated GlcA). Although found throughout the body, CS is a major component of ECM (i.e., bone, cartilage, and central nervous system) and is an essential component of PGs (CSPGs) such as aggrecan, versican, and neurocan. The different sulfation patterns confer different roles to CS and allow selective interactions via electrostatic interactions, with positively charged platelet-derived growth factors (PDGFs) fibroblast growth factor (FGF), insulin-like growth factor (IGF), vascular endothelial growth factor (VEGF), and TGF-β, resulting in the stabilization of these growth factors in solution [59]. CS participates in tissue remodeling and homeostasis and exerts anti-inflammatory activity in articular tissues by reducing proinflammatory factors [60]. Overexpression of CS contributes to chronic inflammatory diseases, including skin lupus erythematosus and dermatomyositis or pulmonary fibrotic diseases [61,62]. Dermatan sulfate (DS) is a stereoisomer of CS and formerly named CS-B. DS chains consist of alternating IdoUA-GalNAc units with 50–200 repeats. Sulfation occurs at the C2 and C4 on IdoUA and C6 on GalNAc residues, respectively. The presence of IdoA residue in DS, like in HS and Hep, appears to play a key role in GAG-binding proteins and particularly with chemokines and cytokines, including IL-8, macrophages inflammatory peptides (MIP-1α and β), RANTES (regulated on activation of normal T cell expressed and secreted), and IFN-γ (for review: [63]). DS interacts as well with several other molecules such as growth factors (FGF family), heparin cofactor II, and ECM components. DS is expressed ubiquitously in ECM and is linked to core proteins (DSPGs) such as decorin, biglycan, versican, thrombomodulin, and endocan. DS has a physiological role in anti-coagulation, wound healing, and tissue development but also participates in pathological processes such tumorigenesis and infection [64]. Maroteaux–Lamy syndrome (MPS-VI) is characterized by a deficiency of N-acetylgalactosamine-4-sulfatase that results in the storage of DS and C4-S [65] (Figure 1). Skeleton, bone, and joints are commonly affected. Other progressive somatic deteriorations are reported with age, like in Hurler syndrome, with the exception that the CNS is spared, as HS is not elevated. Features include coarse facies, enlarged tongue, and corneal clouding, among other features. In the severe form of the disease, MPS-VI patients mostly die before the second decade of life due to cardiac and valvular diseases, pulmonary infection, or restrictive lung diseases. Heart disease and airway obstruction are also major causes of early death in MPS-VII patients, following HS, DS, and CS accumulation [66].

#### 3.1.3. Keratan Sulfate 

Keratan sulfate is a β-1,4-linked Gal and *N*-GalNAc, with sulfate residues can be found on the 6-positions of both residues. KS is the only GAG type without acidic residue. KS is found in cornea (the richest source of KS in the human body), tendon, cartilage, bone, and peripheral nervous systems (for review: [67]). Like HS and DS, KS participates in tissue hydration, cellular recognition of protein ligands, and cell motility. There are two forms of KS (KS I and II), depending on the nature of their linkage to protein [68]. KS chains are generally found structurally attached to a protein core forming proteoglycans (KSPGs) including lumican, keratocan, mimecan, osteomodulin, osteoadherin, and fibromodulin. In cornea, the high abundance of KS appears to play a pivotal role in matrix assembly, which is involved in vision acuity [69]. KS, a major component with CS of aggrecan, is also important for maintaining the proper hydration levels in skeletal tissues, conferring resistance to mechanical stress. Other KSPGs (e.g., ABAKAN, claustrin, PG-1000, phosphocan-KS) are present in neural tissues and interact with several nerve regulatory proteins, suggesting the potential role of KS in axonal guidance and neural angiogenic processes [70]. In MPS-IVA/B (Morquio syndrome), deficiency in the galactose 6 sulfate sulfatase (GALNS) and/or β-galactosidase (Figure 1) impairs the further steps of KS catabolism, which results in abnormal KS and C6-S levels in tissues [71,72]. KS concentrations correlated with clinical severity; in particular, KS accumulation in chondrocytes leads to a systemic skeletal dysplasia [73]. Extra-skeletal manifestations include respiratory impairment, sleep apnea, tracheal obstruction/narrowing, hepatomegaly, heart valve disease, hearing loss, corneal clouding, and dental hypoplasia. Although there can be cervical spinal cord compression, abnormal cognitive development is not affected in most MPS-IV cases, contrary to MPS-I, -II, -III, and -VII. 

Recently, the six mammalian GAGs (i.e., Hep/HS, CS, DS, KS, and HA) have been reported to bind to more than 800 proteins [74]. While Hep and HS, which are the most extensively studied GAGs, interact with many of the proteins (580), followed by KS and CS with 218 and 72 proteins, respectively, a few ligands bind to HA and DS (43 and 19, respectively). Accumulated GAGs in brain, bone, cartilage, and ECM induces pro-inflammatory factors (e.g., TNF-α, RANTES, IL-1, 2, 5), which leads to the dysregulation of several molecules, including degradative proteases (e.g., MMPs, serine proteases, and cysteine cathepsins), and subsequently to chronic disorders [75]. In addition, GAGs are known to play a key role in the regulation of cysteine cathepsins with diverse effects particularly in in the folding, stability, and activity of proteases. These proteases have received much attention for their diverse roles in physiological and pathological processes, and some of them are very attractive molecular targets for therapeutic interventions (extensively reviewed in [5,76,77,78,79]). In the next section, we summarize some of the major characteristics of cysteine cathepsins, their expression/activity in MPS, and their regulation by GAGs.

## 4. Cysteine Cathepsins

### 4.1. Overview

Human cysteine cathepsins are lysosomal proteases belonging to the papain-like cysteine protease family (Clan CA, family C1, [80]). There are eleven cysteine cathepsins encoded in the human genome (cathepsins B, C, F, H, K, L, O, S, V, X, and W). Of note, the mouse genome contains only ten of the human orthologs and does not express cathepsin V. The three-dimensional structure of human cysteine cathepsins have been solved, except for cathepsins O and W. Cysteine cathepsins are monomeric proteins in the 20–35 kDa range, except for cathepsin C, which is a highly conserved tetrameric peptidase (~200 kDa). Cysteine cathepsins share a similar structural scaffold, consisting of two subdomains termed the L- and R-domains (left and right, respectively), according to the standard orientation. The active site, which contains the conserved catalytic dyad Cys25 and His159 (papain numbering) is located between the two domains, at the top of the molecule. Cathepsins require acidic pH for their optimum activity. Neutral or alkaline pH induces the rapid and irreversible inactivation of most cysteine cathepsins, except for cathepsin S [81,82]. All cathepsins consist of a signal peptide, a propeptide, and a catalytic domain, which corresponds to the mature form of the fully active enzyme. After being addressed in the endoplasmic reticulum (ER), the peptide signal is cleaved off while the propeptide is removed in the acidic environment of endosomal/lysosomal system, facilitating the release of the mature form [83]. 

Most lysosomal cysteine cathepsins are ubiquitously expressed in the human body, while some of them (cathepsins K, F, S, V, and W) are tissue- and cell-type specific, suggesting specialized cellular functions [83]. Cathepsin K is found predominantly in osteoclasts (multinucleated cells of bone) and synovial fibroblasts and plays a crucial role in bone remodeling by degrading efficiently type-I and -II collagen fibers. Cathepsin K is also expressed in epithelial cells and fibroblasts. Cathepsins S, F, and V are highly expressed in antigen presenting cells (macrophages, dendritic cells, thymic cortical epithelial cells) and are involved in antigen processing and presentation. Both cathepsins S and V display a potent capacity to degrade elastin fibers. Cathepsin W is highly expressed in natural killer cells and cytotoxic CD8^+^ T-cells, localized mainly to the ER, where it may have specific functions in T-cell cytolytic activity. They are involved primarily in many processes in lysosomes such as nonspecific bulk protein degradation and turnover, antigen processing, prohormone activation, and autophagy. However, cathepsin functions are not limited to the endolysosomal environment. They have been found in cell nucleus but also may be released into the cytoplasm or the pericellular space upon stimulation or cell damage [77,78]. They participate in cell signaling, protein processing, and the degradation of several ECM components (i.e., collagenolytic and elastolytic activities) and have been implicated in normal processes of cell growth and tissue remodeling. 

In addition to the dysregulation of their enzymatic activity, the extra-lysosomal localizations of cysteine cathepsins are usually related to pathological disorders [84]. Cysteine cathepsins have been identified as key proteases in a wide range of diseases including cancer, muscular dystrophy, hepatitis, rheumatoid arthritis, cardiovascular and bone diseases, lung diseases, immune system-related disorders, and neurodegenerative diseases, several of which are associated with chronic inflammation [5]. For example, the selective inhibition of cathepsin K is thought to be beneficial for the treatment of osteoporosis, bone cancers, and certain forms of arthritis, based on the cathepsin K overexpression associated with these diseases [85]. On the other hand, inhibition of cathepsin S significantly decreases the response to antigens, and cathepsin S has been proposed as a therapeutic target for diabetes and certain auto-immune diseases such as asthma and psoriasis [86]. Findings that lysosomal leakage of cathepsin B to the cytosol induces neurodegeneration in related brain disorders may hold promise for therapeutic interventions [87]. Of note, the dysregulation of cysteine cathepsins and their putative roles have been reported in different inherited lysosomal storage disorders, including MPS (for review: [4]). 

### 4.2. Cysteine Cathepsins in Mucopolysaccharidosis

#### 4.2.1. Brain 

**Figure 2:**
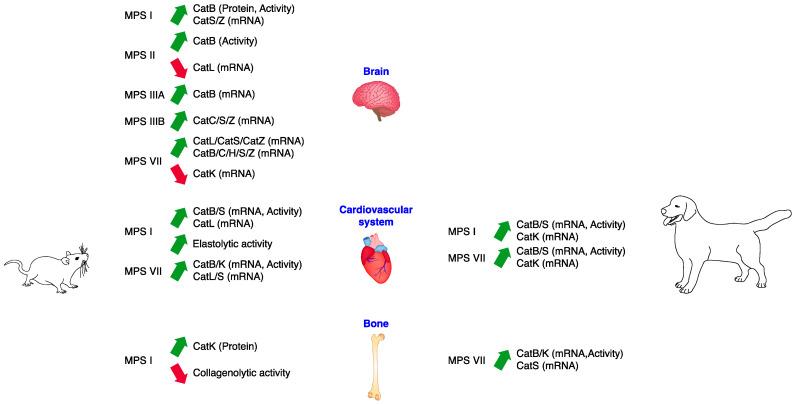
Cathepsins (Cat) expression (mRNA, protein) and activity in MPS animal models. Green and red arrows imply an increase or a decrease of the mRNA, protein and/or activity level, respectively.

As illustrated in Figure 2, high levels of cathepsins B, S, and Z transcripts were found in the cortex of MPS-I and -IIIA/B mouse models [88,89,90]. Overexpression of cathepsin B is associated with an increased deposition of amyloid plaques in the brain of MPS-I mice [91]. Cathepsin B may participate in the inflammasome-dependent pathway involved in neuroinflammation observed in the different types of MPS [92], while both cathepsins B and S may contribute to the progression of neurodegeneration in MPS-I and -IIIB [88,90]. An increase of cathepsin B activity was observed in the brain tissue of MPS-II mice [93]. Cathepsin B inhibition prevented neuronal death and behavioral disorders in a patient with Niemann–Pick type A disease and in an acid sphingomyelinase knockout mouse model [94]. These results suggest that specific cathepsin B inhibition may have neuroprotective effects in MPS patients with neurological disorders. In Hunter syndrome, an analysis of the brain transcriptome of MPS-II mice showed an increase of cathepsins S and Z mRNA expression, with variable expression levels depending on the regions of the brain [95]. Conversely, brain RNA-seq profiling in MPS-II mice indicated a downregulation of cathepsins C, H, L, and S depending on the brain regions [96]. On the other hand, cathepsins B, C, H, S, and Z were overexpressed in all brain regions of MPS-VII mice, while the transcriptional level of cathepsin K was down-regulated in the brainstem [97], suggesting that different cathepsin-related neuropathological mechanisms may predominate in different regions of the brain.

#### 4.2.2. Heart

Cathepsin B was overexpressed in the heart of MPS-I animal models compared to controls, suggesting that the progressive heart failure and valvular disease may be related to an overexpression of the protease [98,99,100]. Treatment of MPS-I mice with a selective cathepsin B inhibitor (CA-074 Me) reduced aortic dilatation and heart valve thickening, leading to cardiac function improvement [101]. Elevated cathepsin B activity was also detected in an MPS-VII dog model with structural collagen abnormalities at the mitral valve [102]. Intravenous injection of a retroviral vector expressing canine β-glucuronidase (the enzyme deficient in MPS-VII) decreased cathepsin B activity and restored collagen structure. Similarly, it has been shown in MPS-I and -VII animal models that aortic dilatation was also associated with the overexpression of cathepsins B, L, K, and S, which possess elastolytic activity [99,103,104]. 

#### 4.2.3. Bone 

Brömme and colleagues previously investigated a murine MPS-I model in which the α-L-iduronidase gene is mutated resulting in an accumulation of DS and HS sulfates [105]. The study reported thickened, shortened bones, and disorganized growth plates with an increased presence of cartilage mimicking that of cathepsin K-deficient mice or patients with pycnodysostosis, a rare genetic disease due to inactivation of cathepsin K. Although the protein level of cathepsin K was higher in MPS-I bones than controls, cathepsin K-related collagenolytic activity was reduced. The large accumulation of GAGs in bone has an inhibitory effect on the collagenolytic activity of cathepsin K, resulting in osteoclast activity impairment and a decrease in bone and cartilage resorption, contributing to bone disorders (see for review: [106]). Upregulation of cathepsins K, B, and S, as well as their peptidase activity, were reported in intervertebral discs of MPS-VII dogs, possibly associated with the induction of Toll-like receptor 4 by GAGs (HS), which in turns activates inflammatory response via the NFκB pathways [107]. 

#### 4.2.4. Lungs 

**Figure 3:**
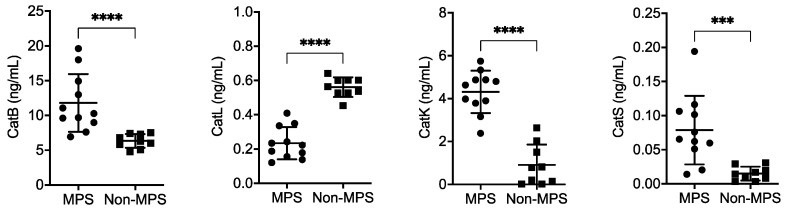
Human cathepsins levels in MPS and non-MPS respiratory specimen. Respiratory specimens, including sputum and tracheal aspirates were collected from MPS patients (N = 11, with 2 MPS-I, 5 MPS-II, and 4 MPS-III) and non-MPS patients (N = 9). Clinical features of patients included in the study were detailed in a previous report [58]. Samples were aseptically weighed and instantly diluted at 1 g/10 mL in a preservative buffer (final concentrations: 100 mM sodium acetate, pH 5.0 plus the peptidase inhibitors 0.5 mM PMSF, 0.5 mM EDTA, 40 µM pepstatin A, and 1 mM MMTS). Samples were then centrifuged for 10 min at 5000× *g* at 4 °C, and the resulting cell-free supernatants were collected, aliquoted, and stored at −80 °C. The total protein quantification of supernatants was performed by BCA assay (ThermoFisher Scientific, Illkirch, France). Protein levels of cathepsins B, L, S, and K (CatB, CatL, CatS, and CatK, respectively) were performed using specific ELISA kit (R&D Systems Europe, Abingdon, UK; and Novus Biologicals, Bio-Techne SAS, Noyal Chatillon Sur Seine, France). Assays (duplicate) were repeated at least three independent times. Statistical analyses were performed using Mann–Whitney U test (***: *p* < 0.001; ****: *p* < 0.0001).

In secretory specimens from MPS-I, -II, and -III patients, we recently reported that levels of cathepsin V, which is the most potent elastase described so far in mammals [108], compared to that of non-MPS patients [58]. Nevertheless, the elastolytic activity of cathepsin V was strongly inhibited by HS from lung biological samples of MPS-I, -II, and -III patients in a dose-dependent manner. Conversely, cathepsin V activity can be restored by an HS antagonist. Molecular modeling studies indicated that HS tetrasaccharide models bound preferentially within the active site of the enzyme. While the overall activity of cysteine cathepsins was reduced in MPS patients compared to non-MPS patients, their activity correlated negatively with HS levels and the global respiratory symptoms severity (GRSS) score, supporting the central role of cysteine cathepsins in lung homeostasis [58]. We also found that cathepsins B, K, L, and S levels were elevated in MPS patients (type I, II, and III), except for cathepsin L, for which concentration was reduced (Figure 3). Of note, cathepsin L participates predominantly in the processing and activation of proheparanase to heparinase (HPSE1), the first enzyme responsible of HS catabolism by cleaving between glucuronic acid and N-acetylglucosamine residues [109]. It can be hypothesized that cathepsin L down expression in MPS may reduce proheparanase processing, favoring the presence of long HS fragments.

### 4.3. Modulation of Cysteine Cathepsin Activity by GAGs

The regulation of lysosomal cathepsins activity by GAGs was first described by Avila and his colleagues [110]. As seen previously, cysteine cathepsins are established as major players alongside other proteases (i.e., matrix metalloproteinases, serine, and acidic proteases,) in extracellular proteolysis. Their action in the GAG-rich extracellular environment raised questions about the interplay between cysteine cathepsins and GAGs outside the lysosome. Depending on the nature, the size of the repetitive disaccharide units, and the pattern of sulfation, GAGs bind to cathepsins at different areas and are important regulators of cysteine cathepsins, particularly in the processing of their proform, stability, and activity of mature cathepsins B, K, S, and V (for review: [6,111]). 

Sulfated GAGs accelerated the autocatalytic processing of procathepsin L but also enhanced proteolytic activities of the full proform toward EMC components [112,113]. This activation mechanism was reported later for procathepsin S [114], procathepsin X [115], and procathepsin B [116]. Long chains and smaller fragments of GAG can both disrupt the interaction between the propeptide and the mature enzyme, thus facilitating automaturation [116]. In the presence of high concentration of C4-S (>0.15%, *m*/*v*), the autoprocessing of procathepsin S into its active mature form was reduced [117].

**Figure 4:**
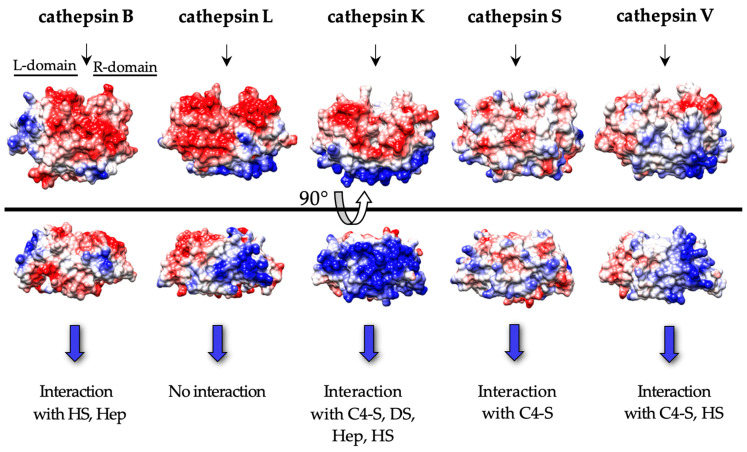
Electrostatic surface potential of cathepsins B, L, K, S, and V. Surface potential representation of cathepsins B (pdb: 1HUC), L (pdb: 2NQD), K (pdb: 1C9E), S (pdb: 1NPZ), and V (pdb: 1FH0) was generated with UCSF Chimera software. The surface colors are fixed at red (−10) or blue (+10); negative charges are shown in red and positive charges are shown in blue. Active site is represented by a black arrow.

Hep and HS also play a major role in the pH stability and activity of mature cathepsin B at neutral pH and alkaline pH [118]. Two putative GAG-binding sites with basic residues were identified in the L- and R-domains of cathepsin B, likely protecting the enzyme from alkaline pH-induced inactivation. While the overall fold of cathepsins is highly conserved, differences in their electrostatic potential molecular surface are observed (Figure 4). 

Especially, cathepsin K possesses a high density of positively charged residues (Lys, Arg) located opposite to the active site, in comparison to the other related cathepsins (Figure 4). Brömme and colleagues have pioneered research on cathepsin K and GAGs. They identified critical positive residues of cathepsin K that interact specifically with negatively charged C4-S, favoring its stability at acidic pH, but also demonstrated the formation of potent active collagenolytic C4-S-cathepsin K complex, unique among mammalian proteases [119,120,121,122,123]. The GAG-binding sites (exosites) are located on the back of cathepsin K, away from the active site. GAGs have been reported as allosteric regulators of cathepsin K, leading to discrete conformational change of the enzyme, favoring its activity and stability [124]. In the absence of C4-S, cathepsin K has only a residual collagenolytic activity against type I and II collagens. Conversely, no effect of GAGs (C4-S, C6-S, DS, and HA) was observed with the closely related cathepsin L, neither on its activity or pH stability. In contrast, a cathepsin L mutant with the active site of cathepsin K (S2 pocket) and the equivalent surface that interacts with C4-S acquired collagenolytic activity in the presence of C4-S, such as wild-type cathepsin K [125]. This mode of regulation received more attention with the discovery that C4-S from cartilage prominently increased the collagenolytic activity of cathepsin K. Due to its central role in bone turnover, cathepsin K is considered one of the most promising targets for the treatment of osteoporosis [85]. DS and HS are able to form complexes with cathepsin K, but are collagenolytic inactive [105]. The concentration of GAGs appears to be important, as high concentrations of HS and DS (>0.15%, *w*/*v*) have an inhibitory effect on the collagenolytic activity of C4-S-cathepsin K complex in vitro. In MPS-I mice, accumulation of HS and DS directly inhibited also the collagenolytic activity of the enzyme, which may impair osteoclast function and contribute afterwards to the skeletal disorders observed in MPS-I [105]. In addition, elastolytic activity of cathepsin K is impaired in the presence of high concentrations of GAGs (C4-S, C6-S, DS, and Hep) [108]. This result suggests that the degradation of extracellular matrix proteins by cathepsin K is efficiently controlled by GAGs, and this may be disturbed in MPS patients. Apart from cathepsin K, proteolytic activities of cathepsins V and S are regulated by sulfated GAGs [58,108,117,121]. As mentioned previously, elastolytic activity of cathepsin V was inhibited by HS in MPS samples in a dose-dependent manner [58]. Similar results were reported with other GAGs (C4-S, C6-S, DS, and Hep) [108]. At the concentration that is found in MPS tissues [126,127], C4-S and to a lesser extent HS act as inhibitors of cathepsin S in vitro toward collagen IV [117]. Docking studies on cathepsin S with C4-S tetrasaccharide identified three putative C4-S-binding sites, which differ from those in the C4-S-cathepsin K complex. One binding site is in the active site and the two others are on the back of the enzyme, one being relatively close to an exosite that was identified to be major in the hydrolysis of elastin [128]. Upon GAG elongation, no proportional increase in docking score was observed. Elastin Congo Red assays revealed that longer GAG chains (C4-S, 0.15%) had little or no effect on the elastolytic activity of cathepsin S, contrary to that observed for cathepsins K and V, which were both inhibited in a dose-dependent manner in vitro [108]. Further studies are needed to explore whether GAGs present in MPS tissues modulate or not elastolytic activity of cathepsin S and contribute to ECM substrates accumulation, promoting progressive symptoms observed in MPS patients, especially cardiovascular and airway disorders. 

It should be noted that GAGs can affect the protease/antiprotease balance [39], for which dysregulation may be crucial in the manifestation of several diseases, including MPS. Recently, we showed that the activity of cystatin C (CC), a potent extracellular low-molecular-weight basic-protein inhibitor of cysteine cathepsins was reduced in vitro by HS in a dose-dependent manner [129]. Consistently, it could be assumed that the impairment of CC inhibitory potential toward cathepsin L found in MPS-I, -II, and -III respiratory specimens [129] may similarly modulate the proteolytic activity of related cysteine cathepsins in other organs, which in turn can contribute to the development of MPS symptoms.

## 5. Conclusions

This review reports the influence of MPS-associated GAGs on expression and proteolytic activities of several cysteine cathepsins in brain, cartilage/bone, cardiovascular, and lungs. The levels of GAGs, the nature and the size of the repetitive disaccharide units, and the pattern of sulfation differently modulate the processing, stability, and activity of specific cathepsins, which in turn alter directly lysosomal, cellular, and extracellular homeostasis in MPS. Developing strategies to reduce GAG contents in MPS and to restore physiological proteolytic activities of cathepsin K, S, and V represent challenging therapeutic avenues for MPS.

## Figures and Tables

**Table 1:** General characteristics of MPS.

MPS Type (Eponym)	Enzyme Deficiency	Gene	Accumulated GAGs	Available Therapeutic Approaches
**MPS-I** (Hurler; Scheie; Hurler-Scheie syndrome)	α-L-iduronidase	*IDUA*	DS, HS	ERT, HSCT
**MPS-II** (Hunter syndrome)	Iduronate-2-sulfatase	*IDS*	DS, HS	ERT, HSCT
**MPS-IIIA** (Sanfilippo syndrome)	Heparan-N-sulfatase	*SGSH*	HS	Not available
**MPS-IIIB** (Sanfilippo syndrome)	N-acetyl-α- glucosaminidase	*NAGLU*	HS	Not available
**MPS-IIIC** (Sanfilippo syndrome)	Acetyl CoA glucosamine N-acetyltransferase	*HGSNAT*	HS	Not available
**MPS-IIID** (Sanfilippo syndrome)	N-acetylglucosamine- 6-sulfatase	*GNS*	HS	Not available
**MPS-IVA** (Morquio A syndrome)	N-acetylgalactosamine-6-sulfatase	*GALNS*	KS, CS	ERT, HSCT
**MPS-IVB** (Morquio B syndrome)	β-galactosidase	*GLB1*	KS	ERT, HSCT
**MPS-VI** (Marotaux-Lamy syndrome)	Arylsulfatase B	*ARSB*	DS, CS	ERT, HSCT
**MPS-VII** (Sly syndrome)	β-glucuronidase	*GUSB*	DS, CS, HS	ERT, HSCT
**MPS-IX** (Natowicz syndrome)	Hyaluronidase	*HYAL1*	HA	Not available

ERT, enzyme replacement therapy; HSCT, hematopoietic stem cell transplantation.

**Table 2:** General characteristics of GAGs (adapted from [41,42]).

GAGs	Disaccharide Units	Degree of Sulfation per Disaccharide (Average)	Molecular Weight (kDa)	Site of Synthesis
**Heparin** **(Hep)**	L-IdoA-α(1→4)—D-GlcNAc-α(1→4) 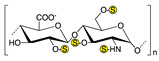	2.5	10–20	Mast cells (liver, lungs, skin)
**Heparan sulfate** **(HS)**	D-GlcA-β(1→4)—D-GlcNAc-α(1→4) 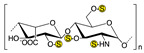	0.8	10–100	Ubiquitous ECM, BM, cell surfaces
**Chondroitin sulfate** **(CS)**	D-GlcA-β(1→3)—D-GalNAc-β(1→4) 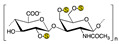	0.9	5–50	Most abundant GAG in the body (cartilage bone, tendon, ligament, aorta, brain, skin)
**Dermatan sulfate** **(DS)**	L-IdoA-α(1→3)—D-GalNAc-β(1→4) 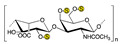	1.1	15–40	Skin, blood vessels, heart valves, tendons, cartilages, lungs, cornea, umbilical cord
**Keratan sulfate** **(KS)**	D-Gal-β(1→4)—D-GalNAc-β(1→3) 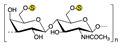	1	4–19	Cartilage, bone, cornea, brain, skin, embryonic liver, lung
**Hyaluronan** **(HA)**	D-GlcA-β(1→4)—D-GlcNAc-α(1→4) 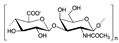	0	4–8000	Non-covalently attached in the ECM, synovial fluid, ECM loose connective tissue, cartilage, skin, brain

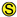
: Possible site of sulfation.

## Data Availability

Not applicable.
